# Human anti-HIV IgM detection by the OraQuick *ADVANCE*® Rapid HIV 1/2 Antibody Test

**DOI:** 10.7717/peerj.4430

**Published:** 2018-02-28

**Authors:** Geraldine Guillon, Graham Yearwood, Casey Snipes, Daniel Boschi, Michael R. Reed

**Affiliations:** OraSure Technologies Inc., Bethlehem, PA, USA

**Keywords:** IgM detection, CDC, POC, Rapid test, Seroconversion, HIV

## Abstract

The Centers for Disease Control and Prevention (CDC) and many public health jurisdictions continue to advocate for the most sensitive rapid HIV test that is available. Currently, the recommendation is to utilize tests that can detect HIV infection biomarkers within 30 days of infection, when initial immune responses are mounted. The infected patient’s IgM response is often used to detect acute infection within a 20–25 days window after infection. This requirement applies to lab-based testing with automated analyzers and rapid, point of care (POC) testing used for screening in a non-clinical setting. A recent study has demonstrated that POC tests using a Protein A-based detection system can detect samples with predominantly HIV-1 IgM reactivity (Moshgabadi et al., 2015). The OraQuick *ADVANCE*® Rapid HIV-1/2 Antibody Test (OraQuick *ADVANCE*®) also uses Protein A as the detection protein in the antibody-binding colloidal gold conjugate, so it is expected that the OraQuick *ADVANCE*® Test will also detect samples with predominantly IgM reactivity. This report definitively demonstrates that the OraQuick *ADVANCE*® Test can detect IgM antibodies during an acute infection window period of approximately 20–25 days after infection, and is therefore suitable for use in testing environments requiring adherence to current CDC recommendations.

## Introduction

**Figure 1:**
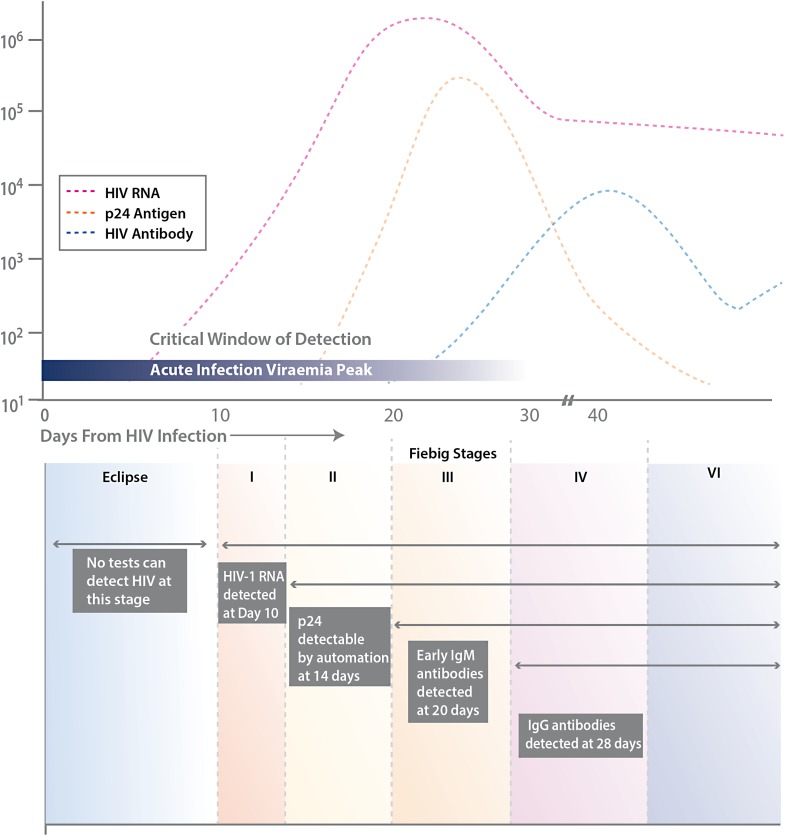
Various biomarkers utilized for HIV detection. Stages of HIV life cycle (Fiebig stages) and relative abundance of biomarkers throughout these stages. Different biomarkers allow for detection of infection at different stages, represented by the boxes and labels below the chart. This figure is adapted from Fiebig et al. (2003).

The Centers for Disease Control and Prevention (CDC) and many public health jurisdictions continue to advocate for the most sensitive rapid HIV test that is available, both to identify positive patients when they are at their most infectious, and to ensure that negative patients are truly negative when starting a pre-exposure prophylaxis regimen (Centers for Disease Control and Prevention and Association of Public Health Laboratories. Laboratory Testing for the Diagnosis of HIV Infection: Updated Recommendations, 2014). Currently, the recommendation is to utilize tests that can detect HIV infection biomarkers within 30 days of infection, when initial immune responses are mounted. Typically, these biomarkers measure the presence of virus, such as p24 antigen or viral RNA, the patient’s IgM and IgG immune response to HIV infection, and/or the level of CD4 T cells (see Fig. 1).

**Figure 2:**
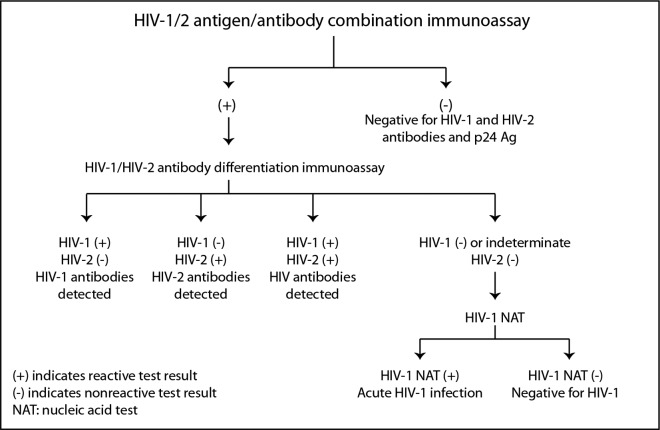
Centers for disease control and prevention HIV testing algorithm. Reproduced from laboratory testing for the diagnosis of HIV infection: updated recommendations, 2014.

The CDC and the Association of Public Health Laboratories (APHL) have issued revised recommendations (Centers for Disease Control and Prevention and Association of Public Health Laboratories. Laboratory Testing for the Diagnosis of HIV Infection: Updated Recommendations, 2014) based on HIV tests approved by the Food and Drug Administration (FDA). The CDC and APHL recommend that laboratories use an automated, laboratory-based p24 antigen/antibody HIV screening immunoassay followed, if reactive, by an HIV-1/HIV-2 antibody differentiation immunoassay. When the differentiation assay interpretation is negative or indeterminate for HIV-1, an HIV-1 nucleic acid test (NAT) is recommended (see Fig. 2).

For those settings where an automated laboratory instrument is not available or serum/plasma sample collection is not practical or cost-effective to utilize, a separate guideline for testing in non-clinical settings has been established (Centers for Disease Control and Prevention. Implementing HIV Testing in Nonclinical Settings: A Guide for HIV Testing Providers, 2016). These guidelines recommend that sites use HIV testing technologies that are the most sensitive, cost-effective, and feasible for use by their agency.

Rapid, POC testing is a logical choice for screening in a non-clinical setting. It is important, for the reasons outlined above, that a POC test is still able to detect infection within the 30-day acute infection window period. The only way that this can occur is by the detection of either the IgM antibody response or p24 antigen (Fiebig et al., 2003). While there is an FDA-approved POC test that detects p24 antigen available today, numerous data has been presented and published showing limited utility of p24 detection in fingerstick whole blood samples due to low sensitivity of the device in the 20–50 pg/ml range compared to automated p24/IgM/IgG assays with sensitivity as low as 0.1 pg/ml (Pebody, 2012; Conway et al., 2014; Lewis et al., 2015). This lack of sensitivity drives the market towards POC tests that detect IgM antibodies.

A recent study has demonstrated that POC tests using a Protein A-based detection system can detect samples with predominantly HIV-1 IgM reactivity (Moshgabadi et al., 2015). The OraQuick *ADVANCE*® Test also uses Protein A as the detection protein in the antibody-binding colloidal gold conjugate. Due to this, it is expected that the OraQuick *ADVANCE*® Test will also detect samples with predominantly IgM reactivity.

The OraQuick *ADVANCE*® Test has proven performance and patient acceptability that is facilitated by specimen flexibility (Wesolowski et al., 2006, Pant Pai et al., 2007, 2012; Zachary et al., 2012, Reynolds & Muwonga, 2014, Nkenfou et al., 2014; Martin et al., 2017). The OraQuick *ADVANCE*® Test is a simple, three-step protocol providing results in 20 minutes. The test is FDA-approved with >99% sensitivity and specificity claimed across many specimen types, including oral fluid, fingerstick blood and whole blood. FDA approval for the OraQuick *ADVANCE*® Test using oral fluid as the specimen type was in 2004, and raw material and manufacturing process changes that allowed earlier detection of seroconversion was FDA approved in 2015.

This report describes the results of a study designed to evaluate the seroconversion performance of the OraQuick *ADVANCE*® Test compared to automated IgG/IgM enzyme immunoassay (EIA) performance and to confirm the hypothesis that its Protein A-based detection system is able to detect samples known to be predominantly IgM positive, thereby demonstrating it is appropriate as a POC diagnostic test for acute HIV infection.

## Materials and Methods

### OraQuick *ADVANCE*® Rapid HIV-1/2 Antibody Test

**Figure 3:**
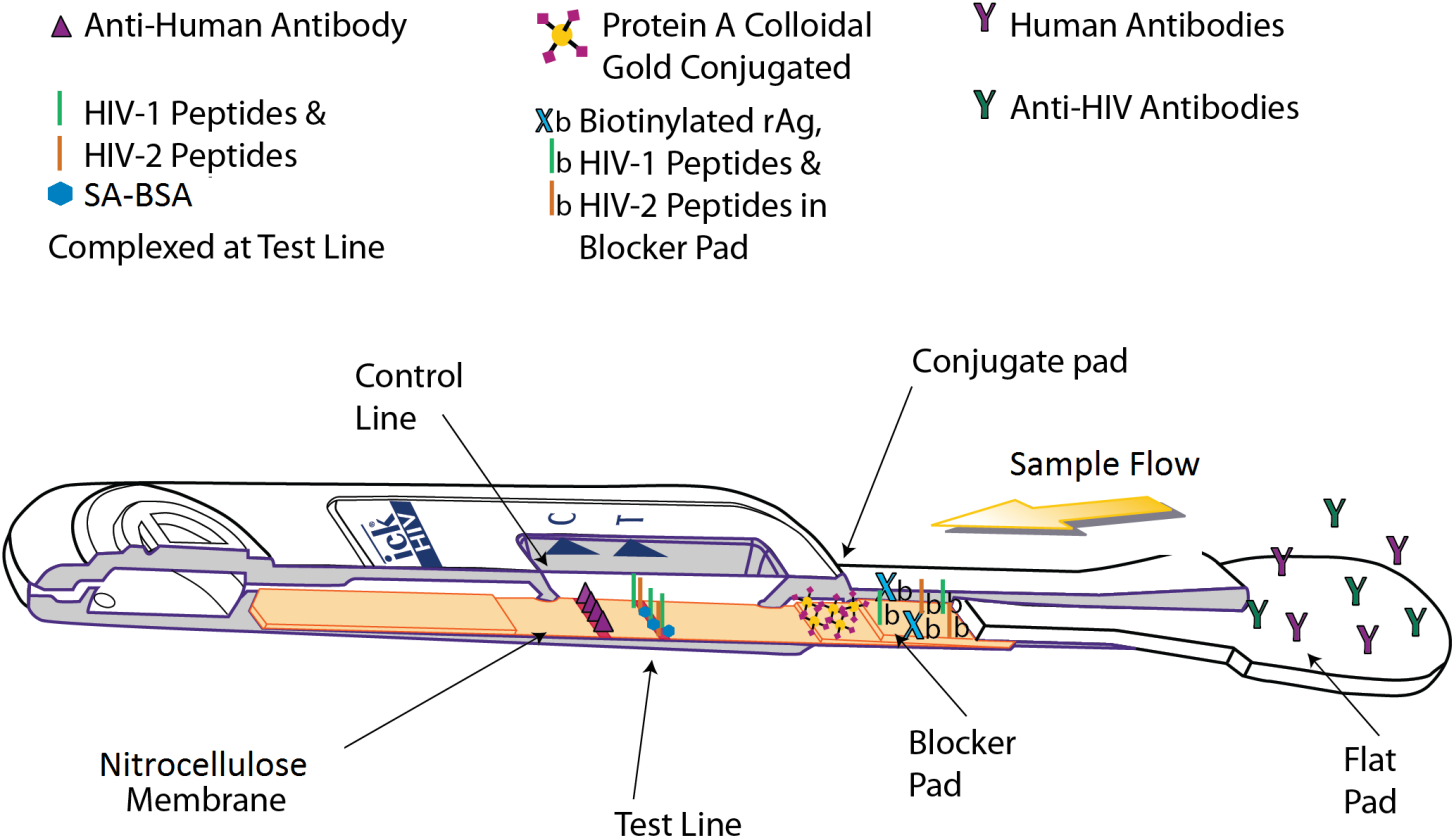
Operating principle of the OraQuick *ADVANCE*® Test. Sample is wicked up the device, hydrating blocker and conjugate pad reagents, which are transferred to the nitrocellulose membrane. HIV antibodies in the sample bind biotinylated reagents and are labeled by Protein A colloidal gold. The immuno-sandwich is immobilized by biotin binding with Streptavidin-BSA, and by anti-HIV antibodies interacting with anchored peptides at the test line. Human antibodies complexed with Protein A colloidal gold are captured at the control line. Accumulation of colloidal gold at the control and test lines creates a reddish-purple stripe.

The OraQuick *ADVANCE*® Test is FDA approved to detect antibodies to human immunodeficiency virus type 1 (HIV-1) and type 2 (HIV-2) in whole blood (from a fingerstick or venipuncture sample), plasma, or oral fluid sample. The assay strip in the device consists of a membrane of nitrocellulose striped with control and test lines. The test line is striped with both HIV-1 and HIV-2 synthetic peptides and modified streptavidin (a biotin-binding protein). HIV-specific antibodies are introduced with the sample, and, if present, will interact with biotinylated HIV-1 and HIV-2 peptides and colloidal gold labeled with Protein A, which are impregnated in different sections of the device. This sample-reagent mixture then flows up the nitrocellulose, with complexed gold particles binding to the test line components in various “sandwich” configurations (Fig. 3).

The presence of HIV-1 and/or HIV-2 antibodies in the tested specimen is indicated by the appearance of a reddish-purple colored band at the test line location of the device within 20–40 minutes.

### Comparison of seroconversion detection by OraQuick *ADVANCE*® Rapid HIV-1/2 Antibody Test to IgG/IgM automated assays

The performance of three independent production lots of the OraQuick *ADVANCE*® Test (lot numbers 15070PL0, 6650009, and 6650592) was evaluated across 201 samples from 23 different commercially available seroconversion panels (Table 1), as described in the package insert. Each panel consists of sequential plasma specimens obtained from a single HIV-infected individual during seroconversion. Five microliters of each seroconversion panel member was spiked into developer solution and three replicates were run per panel member for each lot. Results were read after a 20 minute incubation period. The results were compared to those provided by the seroconversion panel vendor, which were determined using FDA-approved automated IgG/IgM assays.

**Table 1:** Commercially available seroconversion panels used in analysis of seroconversion sensitivity for the OraQuick *ADVANCE*® test.

Vendor	Panel name	Panel number(s)
SeraCare	PRB924	1–8
SeraCare	PRB925	1–6
SeraCare	PRB926	1–6
SeraCare	PRB933	1–3
SeraCare	PRB934	1–3
SeraCare	PRB939	1–9
SeraCare	PRB945	1–6
SeraCare	PRB947	1–4
SeraCare	PRB959	1–7
SeraCare	PRB965	1–6
SeraCare	PRB967	1–6
SeraCare	PRB968	1–10
SeraCare	PRB969	1–10
SeraCare	PRB970	1–4
ZeptoMetrix	6243	1–10
ZeptoMetrix	9015	1–10
ZeptoMetrix	9019	1–3
ZeptoMetrix	9032	1–14
ZeptoMetrix	9076	1–10
ZeptoMetrix	9077	1–29
ZeptoMetrix	9079	1–25
ZeptoMetrix	9084	1–4
ZeptoMetrix	12008	1–13

### Detection of IgM antibodies

A previous study characterized samples in commercially available seroconversion panels as containing detectable levels of either IgG-only, IgG and IgM, or IgM-only by depleting all IgM antibodies from the sample then testing with a reference ELISA and a POC antibody test (Moshgabadi et al., 2015). Due to limited sample availability from the vendor, 15 of the 20 samples identified as being predominantly IgM (greater than 50% of total immunoglobulin content) were utilized in this study. These samples were tested concurrently with the OraQuick *ADVANCE*® Test, and the INSTI® HIV-1/HIV-2 antibody test (INSTI™; bioLytical Laboratories, Richmond, BC, USA) as described in the original report (Moshgabadi et al., 2015), following the package insert in each case.

In this study, these samples were evaluated using the OraQuick *ADVANCE*® Test by spiking 5 microliters of each sample into developer solution. The device was read after a 20 minute incubation period as per the package insert. The INSTI® assay required 50 microliters of sample and the protocol was followed as per the package insert.

## Results

### Comparison to IgG/IgM automated assays

A total of 201 panel members were tested across the 23 seroconversion panels. Each panel member was run in triplicate and a result was determined to be positive with the presence of a complete test line at or above the limit of visual detection in all three replicates (see Table 2). The average performance of the OraQuick *ADVANCE*® Test was compared to the automated EIA data reported in the seroconversion package inserts. Thirteen of the 23 panels had data reported from more than one EIA platform, and in these cases the average of the two or three results was used to compare to the OraQuick *ADVANCE*® Test results. A total of 114 of the 201 panel members tested negative for both EIA and in all three lots of the OraQuick *ADVANCE*® Test. When comparing the overall average of seroconversion detection days after first bleed for EIA to the three lot average, the OraQuick *ADVANCE*® Test detected seroconversion within 1.5 days of the automated IgG/IgM assays (95% CI [−0.1 to 3.1 days]), with three panels being detected earlier by the OraQuick *ADVANCE*® Test, seven on the same day and 13 later.

**Table 2:** Comparison of HIV seroconversion detection between three lots of the OraQuick *ADVANCE*® HIV Test and automated enzyme immunoassay (EIA) tests.

Panel	Days after initial bleed										Days OraQuick *ADVANCE* average–EIA average
	OraQuick 15070PL0	OraQuick 6650009	OraQuick 6650592	OraQuick average	Abbott HIV 1/2 EIA	Abbott HIV AB 1/2 AxSYM EIA	Abbott prism anti HIV 1/2 EIA	Genetic systems HIV-1/2 EIA	Bio-rad HIV-1/2 plus O EIA	EIA average	
924	35	40	35	37	33					33	3.7
925	44	44	44	44	44			49		46.5	−2.5
926	27	27	27	27	27			27		27	0.0
933	27	27	27	27	21			27		24	3.0
934	7	7	7	7	7			11		9	−2.0
939	103	103	103	103	103			103		103	0.0
945	20	20	20	20	13					13	7.0
947	11	20	20	17	9			20		14.5	2.5
959	14	14	14	14	9			14		11.5	2.5
965	12	12	12	12	12					12	0.0
967	19	19	19	19					17	17	2.0
968	33	33	33	33					26	26	7.0
969	70	70	70	70					70	70	0.0
970	14	14	14	14					10	10	4.0
6243	32	32	32	32		32	32			32	0.0
9015	35	35	35	35	35			37	30	34	1.0
9019	38	38	38	38	38			38	38	38	0.0
9032	36	36	36	36	24			51	29	34.7	1.3
9076	74	69	69	71	66				66	66	4.7
9077	45	45	45	45	57				52	54.5	−9.5
9079	55	55	55	55	47				47	47	8.0
9084	49	49	49	49	49					49	0.0
12008	33	35	35	34	33				33	33	1.3
**Average**	**36.2**	**36.7**	**36.5**	**36.5**	**34.8**	**32.0**	**32.0**	**37.7**	**38.0**	**35**	**1.5**
**OQ average for equivalent samples**					**37.3**	**32.0**	**32.0**	**34.8**	**40.9**		
**OQ average–EIA average for equivalent samples**					**2.4**	**0.0**	**0.0**	**−2.9**	**2.9**		

### Detection of IgM antibodies

Among the 15 samples that were characterized as either IgM-only or predominantly IgM (Table 1), 14 of 15 were detectable by both the OraQuick *ADVANCE*® test and INSTI™ (Table 3). As these samples were shown previously to be reactive (Moshgabadi et al., 2015), any sample that was initially non-reactive was repeated in triplicate to confirm the result. The one sample not detected by the OraQuick *ADVANCE*® Test was 943-06 (IgM-only), and the one sample not detected by INSTI® was 938-03 (IgM-only). One sample (950-04—predominantly IgM) was initially scored as non-reactive with the OraQuick *ADVANCE*® Test, but was positive in all three confirmatory tests.

**Table 3:** Detection of predominantly IgM or IgM-only samples by the OraQuick *ADVANCE*® Test and INSTI® test.

Panel name sample	Panel number	% IgM in sample (%)	OraQuick *ADVANCE*® Rapid HIV-1/2 Antibody Test	INSTI® HIV-1/2 antibody test
			Result	Result
PRB914	1	100	R	R
PRB914	2	>50	R	R
PRB914	3	100	R	R
PRB924	8	>50	R	R
PRB925	5	100	R	R
PRB925	6	>50	R	R
PRB927	3	>50	R	R
PRB928	2	100	R	R
PRB934	2	>50	R	R
PRB934	3	>50	R	R
PRB938	3	100	R	NR
PRB940	4	>50	R	R
PRB943	6	100	NR	R
PRB944	5	100	R	R
PRB950	4	>50	R	R

**Note:**

Seroconversion panel members from SeraCare were previously determined to have predominantly IgM or IgM-only HIV antibodies (Moshgabadi et al., 2015). Samples were tested with OraQuick *ADVANCE*® and INSTI® tests and reactivity recorded.

## Discussion

The revised HIV testing recommendations published by the CDC and APHL in June, 2014 have focused testing on early detection during the acute phase of infection (Centers for Disease Control and Prevention and Association of Public Health Laboratories. Laboratory Testing for the Diagnosis of HIV Infection: Updated Recommendations, 2014). Lab-based tests typically use automated p24 antigen/antibody HIV screening immunoassays followed, if reactive, by an HIV-1/HIV-2 antibody differentiation immunoassay. The ability to detect p24 antigen and/or IgM allows these assays to detect the acute phase of infection when patients are most infectious.

It is well established that persons at high risk for HIV infection, such as gay and bisexual males, injection drug users, and minority populations have a history of not engaging in routine care for a variety of system, social and individual factors (Reimen et al., 2015). For these populations, as well as those in rural settings, rapid POC HIV diagnostics have been shown to help identify undiagnosed infections and increase screening in all levels of the healthcare system and outreach programs (Tucker, Bien & Peeling, 2013). It is important that these more convenient tests are also able to detect acute infections, either by p24 antigen or IgM antibody detection.

The performance of the OraQuick *ADVANCE*® Test has been established in clinical population settings to support CE approval in Europe, FDA approval in the US and PreQualification by the World Health Organization. The purpose of the study reported here was to evaluate the ability of the OraQuick *ADVANCE*® Test to (i) detect seroconversion of plasma panels collected from HIV-infected subjects compared to automated lab-based IgM/IgG assays, and (ii) detect HIV antibodies in plasma samples that contain predominantly IgM antibodies to HIV. It is noted that the authors are not aware of any attempts to replicate these results by others.

The results shown in Table 2 demonstrate that the OraQuick *ADVANCE®* Test is able to detect seroconversion an average of 1.5 days earlier than automated assays. While there were differences between platforms, with average differences ranging from 2.9 days earlier (Genetic Systems, Redmond, WA, USA) to 2.9 days later (BioRad, Hercules, CA, USA), 10 of the 23 panels detected seroconversion with the OraQuick *ADVANCE*® Test at the same time or earlier than the automated assays.

It is possible that these panels, and others, would be detected sooner with a test that also includes p24 antigen detection, which highlights a known limitation of antibody-only testing platforms. However, as described above, the only FDA-approved rapid test with p24 antigen does not have sufficient sensitivity in fingerstick whole blood applications (Smallwood et al., 2016), which is preferred for blood-based testing in non-clinical settings, and drives the POC market towards tests that detect IgM antibodies.

Testing of 15 seroconversion samples that were previously characterized (Moshgabadi et al., 2015) as containing predominantly IgM antibodies with the OraQuick *ADVANCE*® Test and INSTI® demonstrated both tests are able to detect IgM antibodies, with each device detecting 14 of 15 predominantly IgM samples, although each failed to detect different samples (Table 3).

## Conclusion

The results of this study demonstrate that the OraQuick *ADVANCE*® Test can detect IgM antibodies during an acute infection window period. Based on this capability it can be inferred that the test is able to detect seroconversion approximately 20–25 days after infection, and is therefore suitable for use in testing environments requiring adherence to the CDC and APHL recommendations.

## Additional Information and Declarations

### Data Availability

The following information was supplied regarding data availability: The raw data is included in the manuscript in the Results section. The raw data is a recording of the visual observation of the presence or absence of a colored line or dot on the devices. As a result, the data presented in the manuscript is a transcription of the raw data recorded by scientists in notebooks.

## References

[ref-1] Centers for Disease Control and Prevention and Association of Public Health Laboratories. Laboratory Testing for the Diagnosis of HIV Infection: Updated Recommendations (2014). https://stacks.cdc.gov/view/cdc/23447.

[ref-2] Centers for Disease Control and Prevention. Implementing HIV Testing in Nonclinical Settings: A Guide for HIV Testing Providers (2016). Center for Disease Control and Prevention. https://www.cdc.gov/hiv/pdf/testing/CDC_HIV_Implementing_HIV_Testing_in_Nonclinical_Settings.pdf.

[ref-5] Conway DP, Holt M, McNulty A, Couldwell DL, Smith DE, Davies SC, Cunningham P, Keen P, Guy R, Sydney Rapid HIV Test Study (2014). Multi-centre evaluation of the determine HIV combo assay when used for point of care testing in a high risk clinic-based population. PLOS ONE.

[ref-6] Fiebig EW, Wright DJ, Rawal BD, Garrett PE, Schumacher RT, Peddada L, Heldebrant C, Smith R, Conrad A, Kleinman SH, Busch MP (2003). Dynamics of HIV viremia and antibody seroconversion in plasma donors: implications for diagnosis and staging of primary HIV infection. AIDS.

[ref-7] Lewis JM, Macpherson P, Adams ER, Ochodo E, Sands A, Taegtmeyer M (2015). Field accuracy of 4th generation rapid diagnostic tests for acute HIV-1: a systematic review. AIDS.

[ref-8] Martin IB, Williams V, Desmond F, Read S (2017). Performance of and preference for oral rapid HIV testing in The Bahamas. Journal of Infection and Public Health.

[ref-9] Moshgabadi N, Galli RA, Daly AC, Ko SM, Westgard TE, Bulpitt AF, Shackleton CR (2015). Sensitivity of a rapid point of care assay for early HIV antibody detection is enhanced by its ability to detect HIV gp41 IgM antibodies. Journal of Clinical Virology.

[ref-10] Nkenfou CN, Kembou JT, Djikeng A, Domkam I, Tchuinkam T (2014). An evaluation of human immunodeficiency virus oral screening test awareness and preferences in the West region of Cameroon. Journal of Infection and Public Health.

[ref-11] Pant Pai N, Balram B, Shivkumar S, Martinez-Cajas JL, Claessens C, Lambert G, Peeling RW, Joseph L (2012). Head-to-head comparison of accuracy of a rapid point-of-care HIV test with oral versus whole-blood specimens: a systematic review and meta-analysis. Lancet Infectious Diseases.

[ref-12] Pant Pai N, Joshi R, Dogra S, Taksande B, Kalantri SP, Pai M, Narang P, Tulsky JP, Reingold AL (2007). Evaluation of diagnostic accuracy, feasibility and client preference for rapid oral fluid-based diagnosis of HIV infection in rural India. PLOS ONE.

[ref-13] Pebody R (2012). More evidence that rapid ‘combination’ test often fails to detect acute HIV infection. NAM Aidsmap. https://www.aidsmap.com/More-evidence-that-rapid-combination-test-often-fails-to-detect-acute-HIV-infection/page/2543010/.

[ref-14] Reimen RH, Bauman LJ, Mantell JE, Tsoi B, Lopez-Rios J, Chhabra R, DiCarlo A, Watnick D, Rivera A, Teitelman N, Cutler B, Warne P (2015). Barriers and facilitators to engagement of vulnerable populations in HIV primary care in New York City. Journal of Acquired Immune Deficiency Syndromes.

[ref-15] Reynolds SJ, Muwonga J (2014). OraQuick ADVANCE rapid HIV-1/2 antibody test. Expert Review of Molecular Diagnostics.

[ref-16] Smallwood M, Vijh R, Nauche B, Lebouche B, Joseph L, Pant Pai N (2016). Evaluation of a rapid point of care test for detecting acute and established HIV infection, and examining the role of study quality on diagnostic accuracy: a Bayesian meta-analysis. PLOS ONE.

[ref-17] Tucker JD, Bien CH, Peeling RW (2013). Point-of-care testing for sexually transmitted infections: recent advances and implications for disease control. Current Opinion in Infectious Diseases.

[ref-18] Wesolowski LG, MacKellar DA, Facente SN, Dowling T, Ethridge ST, Zhu JH, Sullivan PS (2006). Post-marketing surveillance of OraQuick whole blood and oral fluid rapid HIV testing. AIDS.

[ref-19] Zachary D, Mwenge L, Muyoyeta M, Shanaube K, Schaap A, Bond V, Kosloff B, de Haas P, Ayles H (2012). Field comparison of OraQuick® ADVANCE rapid HIV-1/2 antibody test and two blood-based rapid HIV antibody tests in Zambia. BMC Infectious Diseases.

