# Correction: Human anti-HIV IgM detection by the OraQuick *ADVANCE*^®^ Rapid HIV 1/2 Antibody Test

**DOI:** 10.7717/peerj.4430/correction-1

**Published:** 2018-06-22

**Authors:** Geraldine Guillon, Graham Yearwood, Casey Snipes, Daniel Boschi, Michael R. Reed

**Affiliations:** OraSure Technologies Inc., Bethlehem, PA, USA

Corrigendum:

After publication, the authors noticed that the wrong version of Figure 3 was inadvertently published. We have replaced this figure with the correct version. In the correct figure, labels for HIV-1 Peptides, HIV-2 Peptides, and Biotinylated rAg have been removed from the blocker pad and legend.

The authors also noticed a typographical error in the third paragraph of the Discussion section. "... able to detect seroconversion an average of 1.5 days earlier than automated assays" has been amended to "... able to detect seroconversion an average of 1.5 days later than automated assays."

